# Impact of COVID-19 on management of urogynaecology patients: a rapid review of the literature

**DOI:** 10.1007/s00192-021-04704-2

**Published:** 2021-02-03

**Authors:** Jemina Loganathan, Stergios K. Doumouchtsis, CHORUS: An International Collaboration for Harmonising Outcomes, Research and Standards in Urogynaecology and Women’s Health

**Affiliations:** 1 Epsom and St Helier University Hospitals NHS Trust, Dorking Road, Epsom, KT18 7EG UK; 2 St George’s University of London, London, UK; 3 Laboratory of Experimental Surgery and Surgical Research N.S. Christeas, Athens University Medical School, Athens, Greece; 4 American University of the Caribbean, School of Medicine, Pembroke Pines, FL USA

**Keywords:** Coronavirus, COVID-19, Surgical prioritisation, Telemedicine, Urogynaecology

## Abstract

**Introduction and hypothesis:**

The coronavirus (COVID-19) pandemic has impacted health systems worldwide. There is a continuing need for clinicians to adapt practice to facilitate timely provision of medical care, whilst minimising horizontal transmission. Guidance and recommendations are increasingly available, and this rapid review aimed to provide a timely evidence synthesis on the current recommendations surrounding urogynaecological care.

**Methods:**

We performed a literature review using PubMed/Medline, Embase and Cochrane and a manual search of national and international societies for management recommendations for urogynaecological patients during the COVID-19 pandemic.

**Results:**

Nine guidance documents and 17 articles, including 10 reviews, were included. Virtual clinics are recommended for new and follow-up patients, to assess and initiate treatment, as well as triage patients who require face-to-face appointments. Outpatient investigations such as urodynamics and cystoscopy for benign indications can be deferred. Prolapse and continence surgery should be suspended, except in specific circumstances such as procidentia with upper tract complications and failed pessaries. There is no evidence to support a particular route of surgery, but recommendations are made to minimise COVID-19 transmission.

**Conclusions:**

Urogynaecological patients face particular challenges owing to inherent vulnerabilities of these populations. Behavioural and medical therapies should be recommended as first line options and initiated via virtual or remote clinics, which are integral to management during the COVID-19 pandemic. Expanding the availability and accessibility of technology will be increasingly required. The majority of outpatient and inpatient procedures can be deferred, but the longer-term effects of such practices are unclear.

**Supplementary information:**

The online version contains supplementary material available at 10.1007/s00192-021-04704-2

## Introduction

Coronavirus (COVID-19) disease caused by the SARS-CoV-2 virus was first declared as a pandemic by the World Health Organization (WHO) on 11 March 2020 [1]. Since then it has continued to rapidly spread worldwide impacting all aspects of life, not least medical care and how clinicians assess and treat patients. Medical providers worldwide have been required to adapt and streamline services to minimise unwarranted, multiple healthcare facility attendances and patient contact where possible, by conducting remote consultations, delaying non-urgent visits and optimising provision of one-stop services.

The urogynaecology scope of practice involves, to a significant proportion, care and management of elderly and vulnerable patients and therefore these measures are of particular importance. As the pandemic continues, national and international societies and organisations have published guidance for management mainly based on consensus and expert advice given that evidence base to support recommendations is still scarce [2–5].

Rapid reviews are a method of knowledge or evidence synthesis [6] to produce information in a more timely manner than traditional systematic reviews [7]; therefore, they are particularly useful for new and emerging topics. Rapid reviews involve an expedited process with omission of certain steps usually performed in a systematic review.

Given the rapid evolution of evidence, recommendations, policies and clinical management adaptations, a rapid review on the current evidence and recommendations is highly warranted. Since the COVID-19 pandemic was declared, several publications have appeared providing narrative reviews in order to bring all the relevant information from the guidelines together in one document, to support patient care [8–10]. These studies summarise and review published guidelines, original studies, consensus statements, opinions and comments in peer-reviewed journals, and professional organisations and societies.

The aim of this rapid review is to systematically review and evaluate the available evidence from published research, as well as to collate guidelines and recommendations in order to provide guidance on the management of urogynaecological conditions and clinical practices in response to the COVID-19 pandemic. This review has been undertaken by CHORUS, An International Collaboration for Harmonising Outcomes, Research and Standards in Urogynaecology and Women’s Health (i-chorus.org).

## Materials and methods

We performed a literature review using the OvidSP search platform and interrogating through this the databases PubMed/Medline, Embase and Cochrane using keywords and MeSH terms including: COVID-19, SARS-CoV-2, coronavirus, incontinence, pelvic organ prolapse, vaginal prolapse, uterine prolapse, cystocele, rectocele, bladder pain, childbirth trauma, perineal trauma, perineal laceration, urogynaecology, urogynecology, overactive bladder (OAB), recurrent cystitis, recurrent urinary tract infections (UTIs); (Appendix 1).

Literature searches were conducted from 1 January to 22 September 2020. We searched the references of the relevant studies manually using the backward snowballing method [11] in order to identify additional eligible references and studies. In addition, a manual search was conducted of national and international specialist societies and organisations in order to identify practice guidance. We searched the websites of the International Urogynecological Association (IUGA), International Continence Society (ICS), European Association of Urology (EAU), British Society of Urogynaecology (BSUG), Royal College of Obstetricians and Gynaecologists (RCOG), Royal Australian and New Zealand College of Obstetricians and Gynaecologists (RANZCOG), American Urological Association (AUA), American Urogynecologic Society (AUGS), Asia-Pacific Urogynecology Association (APUGA), Urogynecologist Asia (UG-Asia), Urological Association of Asia (UAA), South African Urogynaecological Association (SAUGA) and Pan African Urological Association (PAUSA). The latest version of guidelines was used in cases where more than one guideline or update was available. The final decision about the inclusion of guidelines and published articles was based on authors’ consensus.

All searches were restricted to English-language publications or those with the facility to translate to English, guidelines and best-practice statements. We did not exclude original articles, comments or perspectives. Inclusion criteria were the presence in the articles of guidance or practical advice for the management of urogynaecology patients during the COVID-19 pandemic.

Exclusion criteria were non-English-language articles with translation not readily available, guidelines unavailable to the public in full text, not involving urogynaecology care or not involving urogynaecology care during the COVID-19 pandemic.

Study selection was conducted in stages. Following title screening, the abstracts of all articles in the database were examined. Two reviewers scrutinised the full text of each article and evaluated the studies potentially eligible for inclusion against the inclusion criteria. Discrepancies regarding inclusion or exclusion were resolved through discussion.

Ethical approval was not required for this review. One reviewer extracted relevant data from all eligible articles. The content of each guideline or article was tabulated including the title of the guidance or article, issuing association or journal, and date of publication.

The quality of guidelines was evaluated using the Appraisal of Guidelines for Research and Evaluation (AGREE II) instrument [12] and the quality of reviews assessed using Scale for the Assessment of Narrative Review Articles (SANRA) [13].

## Results

**Fig. 1:**
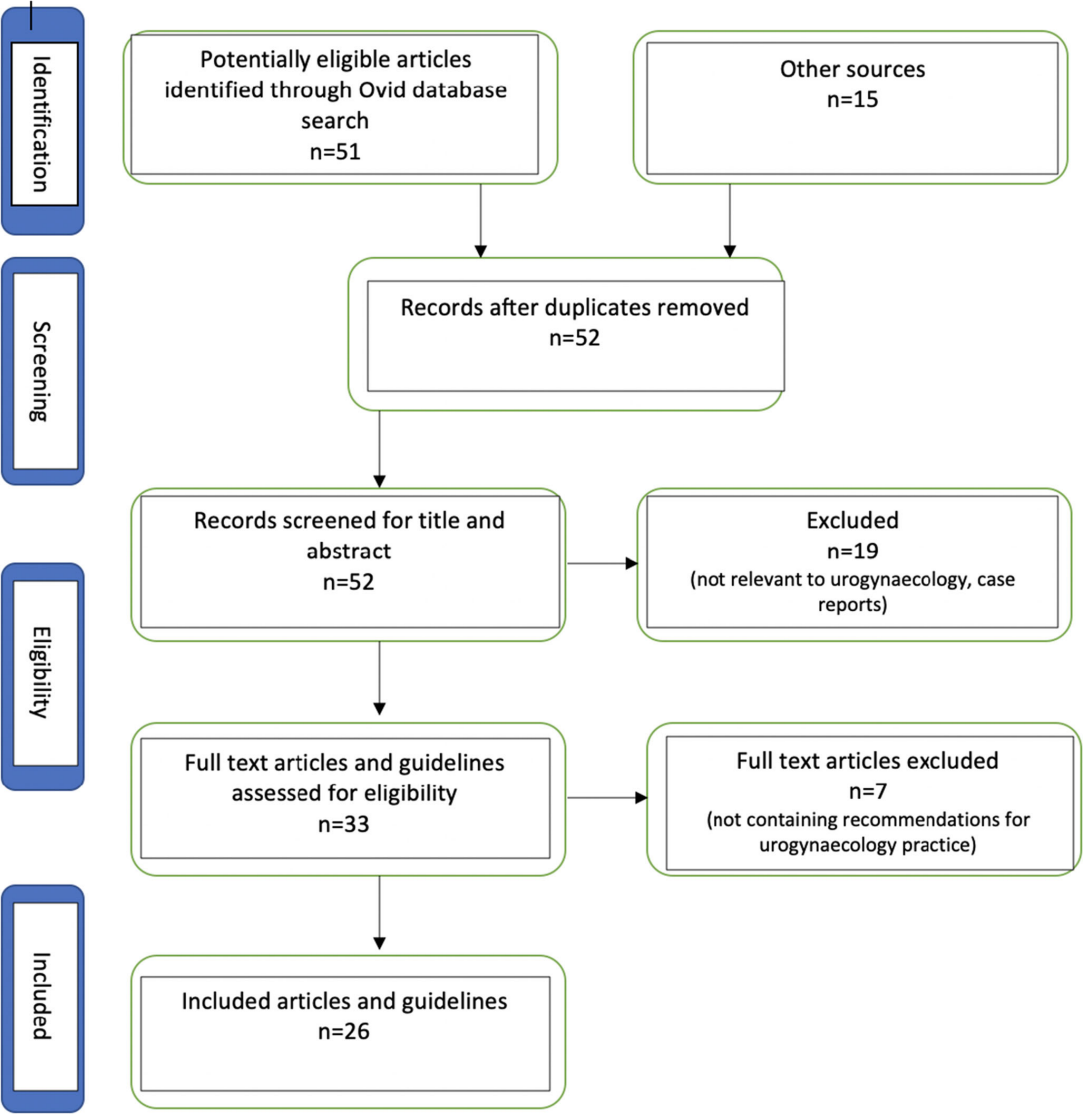
Preferred Reporting Items for Systematic Reviews and Meta-Analyses (PRISMA) diagram

Nine guidance documents and 17 articles, 10 of which are reviews, were included (Fig. 1; Table 1).

**Table 1:** Included articles and guidelines

Reference	Date	Title	Article type	Topics included	Quality assessment score
[14]	23 March	Urology practice during the COVID-19 pandemic	Guidance	Surgical prioritisation	5
				Surgical techniques to minimise exposure	
				Outpatient procedures	
[2]	26 March	Joint RCOG/BSGE Statement on gynaecological endoscopy during the COVID-19 pandemic	Guidance	Laparoscopic and hysteroscopic surgery	4
				Limiting horizontal spread of COVID-19	
				Optimising patient outcomes	
[4]	27 March	Joint Statement on minimally invasive gynecologic surgery during the COVID-19 pandemic	Guidance	Limiting horizontal spread of COVID-19	5
				Endoscopic surgery	
				Vaginal and open abdominal surgery	
[15]	3 April	COVID-19: outpatient services; office consultations and procedures	Guidance	Outpatient clinics and procedures	4
[3]	9 April	BSUG guidance on management of urogynaecological conditions and vaginal pessary use during the Covid 19 pandemic	Guidance	Assessment and management of lower urinary tract symptoms	5
				Management of prolapse	
				Pessary management	
				Outpatient procedures	
[16]	20 April	COVID-19: recommendations for functional urology	Guidance	Assessment and management of lower urinary tract symptoms	6
				Management of prolapse	
				Outpatient procedures	
				Surgical prioritisation	
[17]	28 April	Guidance for the management of urogynecological conditions during the coronavirus (COVID-19) pandemic	Guidance	Assessment and management of lower urinary tract symptoms	6
				Management of prolapse	
				Post-operative follow-up	
[18]	28 April	Joint Statement on re-introduction of hospital and office-based procedures in the COVID-19 climate for the practicing urogynecologist and gynecologist	Guidance	Surgical prioritisation	5
				Inpatient and outpatient procedures	
[5]	July	An organisation-wide collaborative effort to adapt the EAU guidelines recommendations to the COVID-19 era	Guidance	Surgical prioritisation	6
				Management of lower urinary tract symptoms	
[19]	27 April	A guide for urogynecologic patient care utilizing telemedicine during the COVID-19 pandemic: review of existing evidence	Review	Virtual clinics	12/12
				Management of lower urinary tract symptoms	
				Management of prolapse	
				Pessary management	
[20]	24 May	Forecasting the future of urology practice: a comprehensive review of the recommendations by international and European associations on priority procedures during the COVID-19 pandemic	Review	Telemedicine	11/12
				Prioritisation strategies for oncological and non-oncological urology procedures	
				Minimally invasive surgery	
[21]	29 May	Practical recommendations for gynecologic surgery during the COVID-19 pandemic	Review	Surgical prioritisation	8/12
[22]	17 June	Guidance for gynecologists utilizing telemedicine during COVID-19 pandemic based on expert consensus and rapid literature reviews	Review	Telemedicine	12/12
[23]	18 June	Telehealth in urology: a systematic review of the literature. How much can telemedicine be useful during and after the COVID-19 pandemic?	Systematic review	Telemedicine	12/12
				Management of lower urinary tract symptoms	
[8]	23 June	COVID-19 and gynecological cancer: a review of the published guidelines	Review	Reducing horizontal transmission	11/12
				Surgical prioritisation	
				Outpatient clinics	
				Inpatient admissions	
[24]	9 July	Triaging office based urology procedures during the COVID-19 pandemic	Recommendations	Outpatient procedures	
[25]	25 August	How did COVID-19 pandemic change the way we attend the patients in an urogynaecological unit	Review	Assessment and management of lower urinary tract symptoms	9/12
				Management of prolapse	
				Surgical prioritisation	
				Telemedicine	
[26]	2 September	A lasting impression: telemedicine in urogynecology during the coronavirus disease 2019 pandemic	Review	Telemedicine	10/12
[27]	15 September	A systematic review on guidelines and recommendations for urology standard of care during the COVID-19 pandemic	Review	Uro-oncology	12/12
				Endoscopic and robotic surgery	
				Outpatient procedures	
[28]	15 September	Management of female and functional urology patients during the COVID pandemic	Review	Surgical prioritisation	11/12
				Surgical techniques to minimise exposure	
				Management of lower urinary tract symptoms	
[29]	March	Resumption of elective surgery following COVID-19 outbreak, guideline for female pelvic medicine and surgery	Editorial	Surgical prioritisation	
[30]	1 June	Global challenges to urology practice during the COVID-19 pandemic	Comment	Surgical prioritisation	
[31]	11 June	Technology-based management of neurourology patients in the COVID-19 pandemic: is this the future? A report from the International Continence Society (ICS) institute		Virtual clinics	
[18]	1 July	Widespread postponement of functional urology cases during the COVID-19 pandemic: rationale, potential pitfalls, and future consequences	Editorial	Assessment and management of incontinence and voiding disorders	
				Management of prolapse	
[32]	2 July	Virtual consent for virtual patients: benefits of implementation in a peri- and post-COVID-19 era	Editorial	Virtual clinic and consent	
[33]	17 July	Neuro-urology during the COVID-19 pandemic: triage and priority of treatments	Letter to editor	Voiding dysfunction	
				Neurogenic bladder	

*RCOG* Royal College of Obstetricians and Gynaecologists, *BSGE* British Society for Gynaecological Endoscopy, *AUGS* American Urogynecologic Society, *RANZCOG* Royal Australian and New Zealand College of Obstetricians and Gynaecologists, *BSUG* British Society of Urogynaecology, *EAU* European Association of Urology

Quality assessment of guidelines was performed using Appraisal of Guidelines for Research and Evaluation II instrument (AGREEII). Overall assessment scores are shown in Table 1 (1 lowest quality to 7 highest quality). See Appendix 2 for individual domain scores.

Review articles were assessed using the Scale for the Assessment of Narrative Review Articles (SANRA) with a maximum score of 12. See Appendix 3 for the full SANRA scale.

### Recommendations

All 12 articles and guidelines that included outpatient clinic recommendations stated that virtual clinics should be used to minimise horizontal transmission. Virtual clinics can be used for all non-urgent indications such as urinary incontinence and prolapse, and for both initial consultations and follow-up appointments. Patient satisfaction is unaffected and clinic attendance may be increased owing to a reduction in non-attendance [19]. When used for postoperative follow-up there is no increase in adverse outcomes [19]. For patients awaiting surgery, virtual clinics can be conducted to rediscuss alternative therapies.

During virtual clinics, patients can be triaged and limited face-to-face appointments arranged if necessary. When seen face to face, appropriate screening should be undertaken, personal protective equipment (PPE) worn, physical distance maintained, and sanitation available [31].

It has been reported that COVID-19 transmission could be as high as 12.8% at a physical distance of less than 1 m compared with 2.6% at a distance of more than 1 m, reflecting the importance of maintaining physical distance [34].

In keeping with these findings, the Scientific Advisory Group for Emergencies (SAGE), who provide scientific and technical advice to support government decision makers in the UK, reported that COVID-19 transmission could be 2-10 times higher at a physical distance of 1 m compared with 2 m [35]. See Table 2 for a summary of guidance for virtual clinics and inpatient admissions.

**Table 2:** Summary of guidance for virtual clinics and inpatient admissions

Reference	Virtual clinics/telemedicine	Outpatient department, inpatient admissions
[17]	Postoperative follow-up can be virtual	
	Non-inferior for patient satisfaction, complication rates and adverse events	
[31]	Cancel all face-to-face outpatient appointments	
	Virtual consultations where possible	
	Can identify patients requiring urgent consultation	
[28]	Initial and follow-up consultations can be virtual	If seeing face-to-face, patient to wear surgical mask and gloves, clinician to wear apron, surgical mask, visor and gloves
	Triage patients for face-to-face consultation	Invasive tests: clinician wears N95 mask, impermeable gown, gloves and visor
[19]	Virtual clinics: patient satisfaction unaffected, can increase clinic attendance	
	Postoperative virtual clinics: no increase in adverse outcomes or primary care visits	
	Native tissue prolapse repair and mid-urethral sling with no incontinence can be safely followed up in virtual clinic	
	Triage all patients for virtual clinic: established patients not requiring examination, new patients who would benefit from non-surgical treatment, postponed patients awaiting surgery to rediscuss alternative therapies	
	Provide patient information leaflets from established bodies	
[8]	Virtual clinics	Physical distancing
		Sanitisation areas
	Work from home	Limit friends and family accompanying
	Minimise face-to-face	Adequate PPE
[3]	Virtual clinic for pessary follow-up	
	Triage patients: see semi-urgently, within 30 days or delayed review	
[16]	Use telemedicine	
	Avoid face-to-face where possible	
[26]	Telemedicine to minimise exposure	
[22]	Use telemedicine	
	Avoid face-to-face where possible	
	Use telemedicine to assess need for face-to-face review	
	Postoperative follow-up: equal patient-related outcomes with telemedicine compared with face-to-face	
[36]	Use video or teleconsults for all non-urgent indications	
[5]	Use telemedicine to allow physical distancing and minimise footfall	
[18]	Telemedicine whilst awaiting surgery to help with symptom management	
[15]		Essential staff only in clinic rooms
		Discourage accompanying persons
		Physical distancing
		Cleaning surfaces with appropriate disinfectant
		Handwashing before and after patient contact
		Waiting and clinic room with appropriate safe spacing
		COVID-19 positive or those in isolation should not be seen face-to-face. If no option, then wear appropriate PPE
		If face-to-face appointment, screen all patients and accompanying persons for symptoms, travel and exposure

*PPE* personal protective equipment

Of 15 articles and guidelines providing recommendations regarding the management of urinary incontinence and OAB, 12 advise behavioural therapies as the first line.

Two recommend use of smart phone apps to supplement education, for example, for Kegel exercises [19, 26]. Suspension of invasive therapies for urinary incontinence is advised, except where stage 1 sacral neuromodulation is in place or in cases of neurogenic bladder with a high risk of upper renal tract complications [33]. Pelvic floor muscle training is recommended as the first-line for symptomatic prolapse [16, 17, 19, 23, 26]; however, in one editorial, suspension of pelvic floor muscle training is suggested to maintain physical distancing [36].

Use of pessaries is recommended, whilst prolapse surgery is deferred [16, 26, 36], and the pessary change interval can be extended by 3–6 months unless the patient has symptoms of ulceration or fistulation [3, 24, 27]. See Table 3 for a summary of guidance for urinary incontinence and prolapse.

**Table 3:** Summary of guidance for urinary incontinence and prolapse

Reference	OAB, UUI and SUI	Prolapse and pessaries
[17]	Virtual consultations	Virtual consultations
	Use validated questionnaires for urinary symptoms	Use validated questionnaires for prolapse
	Lifestyle measures, PFMT as first line	If mild symptoms: PFMT
	Consider starting antimuscarinics/B3 agonist/vaginal oestrogen	If severely affecting bladder/bowel function and/or ulcer present, may require face-to-face appointment
	Regular follow-up, i.e. 4 weeks after starting antimuscarinics	Pessaries: arrange face-to-face if bleeding or pain symptoms
	Yearly review of long-term antimuscarinic	Can delay pessary change for an additional 3 months after routine 6-month interval, then review
	For SUI consider incontinence pessaries	
[33]	For neurogenic SUI: device implantation can be deferred until safe, no time limit, use pads in interim	
	Erosion from implants requiring removal of prosthesis: defer up to 4 weeks	
	Neurogenic bladder with risk factors for upper renal tract, e.g. DSD: Botox can be deferred up to 8 weeks	
	Neurogenic bladder without risk factors for upper renal tract: can defer Botox during pandemic, no time limit	
[31]	Can teach and monitor PFMT via video consultation	
[28]	Delay all continence procedures until after COVID crisis	
	Manage as outpatients with conservative and medical therapy	
	Delay all new sacral neuromodulation until end of COVID crisis	
	Remove percutaneous nerve evaluation lead in outpatient clinic if one in situ	
	If infected implant treat with intravenous antibiotics; if severe infection remove urgently, i.e. <2 weeks	
	Conservative and medical treatments for SUI and OAB/UUI	
[27]	Stage 2 neuromodulation: no delay owing to risk of infection	Pessary changes: defer for 3–6 months
[19]	Behavioural measures	Virtual consultations
	Self-inserted incontinence tampons or pessaries can be recommended	Online instructions for PFMT
	Patients having invasive treatment, e.g. intravesical Botox, can restart antimuscarinic/mirabegron until service restarts	Behavioural measures, e.g. weight loss, Kegel exercises, PFMT
	Short-term antimuscarinic unlikely to cause dementia therefore can use in elderly if required	Smart phone apps, e.g. for Kegel training
		Home biofeedback devices
		Pessaries: encourage self-cleaning at home
		Can safely delay change up to 6 months
		Consider vaginal oestrogen and empiric treatment for bacterial vaginosis
		If bleeding/discharge can remove and observe for voiding dysfunction prior to clinic review
[3]	Initial virtual consultations	Initial virtual consultations
	Can commence treatment remotely	Procidentia causing bowel/urinary problems need early review within 30 days
	Provide patients with information resources	Pessaries: face-to-face review within 7 days if symptoms suggestive of fistulation
		Pessaries: face-to-face review within 30 days if bleeding/pain/ulceration
		Pessaries: refer via local PMB cancer pathway if PMB with pessary and uterus in situ
		Ring pessaries: can defer change up to 6 months
		Shaatz, shelf, Gelhorn, double pessaries: defer for a maximum of 3 months
		Patients to be given contact numbers in the case of symptoms of ulceration
[16]	Encourage conservative and medical treatments	Virtual clinics
	SUI: all new patients with signs of retention and overflow, see face-to-face for PVR with external probe	If grade 4 prolapse, consider US KUB
		Favour pessary management
		Consider surgery if stage 4 prolapse, failed pessaries and obstructive renal failure
[26]	Non-surgical options as first line whilst elective surgeries restricted	Non-surgical options as first line whilst elective surgeries are restricted
	Medication management	Smart phone apps
	Smart phone apps	
[25]	Start all UI consultations using telemedicine	
	Supplement with use of mobile apps	
	Conservative measures—weight loss, bladder training, PFMT, Kegel exercises ± medications	
[36]	Prescribe medication if required, all intravesical Botox postponed	PFMT postponed to maintain physical distancing
	PFMT postponed to maintain physical distancing	Use pessary
	Screen for red flag symptoms that may indicate bladder cancer and warrant urgent cystoscopy	Urgent surgery if grade 4 prolapse/renal tract complications and failed pessaries
	Postpone SNS unless in test phase. If test phase, consider removal or placement of pacemaker under local anaesthesia	
[21]	Use non-surgical management UI as advised by IUGA	
[23]	Evidence that behavioural measures and PFMT via video conferencing as effective as face-to-face	Use of behavioural measures and PFMT
[5]	Use conservative and medical treatments	
[24]		Can delay pessary change up to 3 months if no erosion or ulcer

*BSUG* British Society of Urogynaecology, *EAU* European Association of Urology, *OAB* overactive bladder, *UUI* urge urinary incontinence, *SUI* stress urinary incontinence, *PFMT* pelvic floor muscle training, *DSD* detrusor sphincter dyssynergia, *PVR* post-void residual volume, *UI* urinary incontinence, *SNS* sacral nerve stimulation, *IUGA* International Urogynecological Association, *US KUB* ultrasound kidneys, ureters and bladder, *PMB* postmenopausal bleeding

Acute retention or a blocked catheter warrants urgent review for catheterisation [20].

If an indwelling catheter is in situ, routine changes can be deferred for 2–4 weeks, unless the patient has a history of difficult changes or recurrent UTIs [24]. Deferring suprapubic catheter changes [3, 20] for up to 3 months has been suggested and changes in the community rather than in the hospital setting are preferred [3, 36].

Urinary tract infections can be managed via virtual consultation [17, 19, 23, 25]. If the patient has recurrent UTIs conservative measures and non-antibiotic therapies should be encouraged [17]. If antibiotics are required, they should be prescribed according to previous culture results. Face-to-face review should be arranged if the patient has complicated UTI or is refractory to treatment [19]. See Table 4 for a summary of voiding dysfunction and urinary tract infection.

**Table 4:** Summary of voiding dysfunction and urinary tract infection

Reference	Voiding dysfunction and catheters	Urinary tract infection
[17]	Severe voiding difficulty requires face-to-face appointment for PVR ± ISC	Virtual consultations
		Acute UTI: consider antibiotics based on symptoms and previous cultures
		For recurrent UTI: non-antibiotics therapies, fluid advice, hygiene advice, vaginal oestrogen low dose. Self start or rotating antibiotics. Safety net re: ascending infection
[33]	If chronic retention, no limit on deferral	
	IDC if ISC not available	
[31]	Can teach and monitor ISC via video consultation	
[28]	If acute retention, see face-to-face to assess for IDC or SPC ± US KUB. Delay functional tests	Conservative and lifestyle measures: hygiene, non-antibiotics therapies, low-dose antibiotics, vaginal oestrogen
	Indwelling catheter: can defer by 4 weeks. Change earlier if encrustations/blockages	
[24]	IDC: can defer change for 2–4 weeks unless history of difficult changes or recurrent UTI	
[19]	Encourage conservative measures to help void	Culture with every episode and treat whilst awaiting results
	Chronic retention >300 ml >6 months and acute retention: face-to-face review	Previous cultures can guide prescribing
	CISC preferable to IDC	Remote prescribing effective, may have a negative impact on antibiotics resistance. Fever and diabetes can indicate severe infection, may warrant face to face appointment
		Prescribing: nitrofurantoin or cotrimoxazole 3–7 days. Seven-day course for the elderly and diabetic. Fluoroquinolone in complicated UTI to avoid admission
		Encourage conservative measures, e.g. cranberry, hydration, d-mannose, vaginal oestrogen
		Consider face-to-face review if refractory UTI with complications
[20]	Acute retention: see face-to-face	
	Defer all SPC and IDC changes	
[14]	Acute retention: see face-to-face for IDC or SPC	
[3]	If acute retention need emergency/urgent review (within 12 hours) for IDC	
	If arranging TWOC, can defer on a case-by-case basis. If high PVR, then teach CISC	
	Change of SPC can be delayed up to 3 months	
	Aim for SPC change in community not hospital setting	
[25]	Encourage conservative measures to help void, e.g. double/triple voiding	Empirical treatment of UTI, including recurrent UTI
	Chronic urinary retention, e.g. >300 ml for >6 months, consider USS KUB and face-to-face consultation for ISC or IDC	Electronic prescribing is effective and efficient
	ISC preferable to IDC	Resolution of symptoms indicative of cure
	Teach ISC face-to-face, follow-up via virtual clinic	
[36]	Acute retention: place IDC or SPC, change regularly in the community. Consider ISC if teaching and education possible	
[30]	Obstructive urinary disorders—face-to-face clinics with reduced capacity	
[5]	Voiding dysfunction: teach ISC or catheterise	Sepsis/complicated UTI: high priority
	Blocked catheter requires emergency review	
[23]		Can be managed safely and effectively using telemedicine

*BSUG* British Society of Urogynaecology, *EAU* European Association of Urology, *PVR* post-void residual volume, *ISC* intermittent self-catheterisation, *IDC* indwelling urethral catheter, *SPC* suprapubic catheter, *US KUB* ultrasound of the kidneys, ureters and bladder, *UTI* urinary tract infection, *CISC* clean intermittent self-catheterisation, *TWOC* trial without catheter

Gross haematuria requires urgent investigation with cystoscopy; however, microscopic haematuria investigations can be deferred. A systematic review of telemedicine in urology, however, reported that data indicate that virtual clinics for initial evaluation are feasible, effective, and associated with a high degree of patient satisfaction [23].

Bladder pain syndrome investigations should be deferred, but oral treatments can be started [5, 28].

Fourteen articles reported recommendations for outpatient procedures, including cystoscopy, intravesical Botox and urodynamics. All urodynamics and cystoscopy for benign indications should be deferred. See Table 5 for a summary of guidance for haematuria, bladder pain syndrome and outpatient procedures.

**Table 5:** Summary of guidance for haematuria, bladder pain syndrome and outpatient procedures

Reference	Haematuria and bladder pain syndrome	Outpatient procedures
[17]	Referral to secondary care if gross haematuria	
[24]	Gross haematuria: urgent cystoscopy, no deferring	Delay urodynamics for 3–6 months
	Microscopic haematuria with risk factors: can defer for up to 3 months unless symptomatic	
	Microscopic haematuria and no symptoms: can defer for 3 months or more	
[27]	Most but not all experts recommend urgent cystoscopy for macroscopic haematuria. EAU and USANZ say it can be deferred for 1–2 months	Delay urodynamics. Time frame 1–6 months
		Neurogenic intravesical Botox can be deferred for up to 4 weeks
		Slings: clinical harm unlikely if postponed for 6 months
[20]	Macroscopic haematuria: urgent cystoscopy	Defer all cystoscopy for benign conditions
	Microscopic: postpone	
[23]	Use telemedicine for initial haematuria consult and triage, then see face-to-face if needed	
[30]	Continue cystoscopy for suspected cancer	All outpatient cystoscopy suspended, continue only for suspected cancer
[28]	Delay BPS investigations until after COVID	Do not commence new intravesical Botox treatments
	Use oral medications, e.g. amitriptyline	Delay intravesical Botox until end of COVID crisis
	Continue bladder instillation if self-administered already	
	Defer if administered in hospital	
[19]	Consider face-to-face review if acute BPS flare requiring instillation	
[5]	Manage BPS conservatively	All urodynamics postponed
	Can offer amitriptyline	
[31]		All urodynamics postponed
		Intravesical Botox can be carried out under local anaesthetic for high-risk patients, e.g. autonomic dysreflexia
[14]		Defer all cystoscopy for benign conditions
[3]		Defer all outpatient treatments and investigations, i.e. cystoscopy (non-cancer indications), bladder instillations, PTNS
[16]		Intravesical Botox suspended unless neurological bladder with upper tract risk
		Cystoscopy: perform within 2 months if risk factors for cancer and refractory OAB
[25]		If planned intravesical Botox, can defer and restart antimuscarinics/B3 agonists
[36]		Intravesical Botox postponed. Consider continuing under local anaesthesia for neurogenic bladder with renal tract complications
[29]		Intravesical Botox: non-essential, i.e., not time sensitive unless, e.g. failure of conservative and progressive symptoms
[18]		Tier 1 can delay beyond 12 weeks i.e. new Botox, new bulking, new PTNS, urodynamics, pessary fittings, new PFMT
		Tier 2 delay 4–12 weeks, e.g. repeat bulking agent, pessary cleaning, PFMT follow-up
		Tier 3 delayed for up to 4 weeks
		Microscopic haematuria, established PTNS, bladder instillations
		Tier 4 cannot be delayed
		Macroscopic haematuria, new ISC instruction, voiding trial, urinary retention, SPC follow-up

*EAU* European Association of Urology, *BSUG* British Society of Urogynaecology, *USANZ* Urological Society of Australia and New Zealand, *BPS* bladder pain syndrome, *PTNS* percutaneous tibial nerve stimulation, *OAB* overactive bladder, *PFMT* pelvic floor muscle training, *ISC* intermitten self-catheterisation

Recommendations regarding surgery advise regional or local anaesthesia where possible, in order to reduce aerosol generation with general anaesthesia [2, 19, 25, 28]. Screening for COVID-19 symptoms and testing preoperatively is advised, as evidence has shown poorer surgical outcomes for asymptomatic COVID-19 patients, therefore surgery may worsen or accelerate progression [2, 4, 5, 8, 14, 21, 28, 30]

Although better able to maintain physical distance and potentially shorter hospital stays with laparoscopic surgery than with open surgery [8], no evidence is available to support a specific route of surgery; therefore, this is at the surgeon’s discretion [5, 20].

Recommendations to reduce horizontal transmission in surgery include having essential staff only in theatre, low electrocautery settings, closed smoke evacuation and minimising blood and fluid droplet spray [4, 5, 8, 14, 16, 20, 27, 28]. See Table 6 for a summary of guidance for elective surgery and techniques to minimise horizontal transmission.

**Table 6:** Summary of guidance for elective surgery and techniques to minimise horizontal transmission

Title of article or guidance	Elective surgery and consent	Surgical techniques to minimise horizontal transmission
[2]	Outcomes worse for asymptomatic COVID-19 patients so surgery may worsen or accelerate progression	No evidence of increased risk with laparoscopy when PPE worn
	COVID-19 test all patients	Vacuum suction devices for desufflation
	14 days self-isolation preoperatively	Use smoke extractor
	Temperature on admission, defer if ≥37.3°C and retest after 14 days	
	Aim for local/regional anaesthetic if possible	
	Negative pressure in theatre	
	High frequency of filtered air exchange	
	Essential theatre staff only	
	Most experienced surgeon operating	
	PPE when GA: water repellent, long-sleeved gowns, eye and face protection, gloves and FFP3 respirators	
	If pyrexial within 30 days screen and retest for COVID-19	
[28]	No contraindications to open, transurethral and vaginal procedures	Low power setting for electrosurgery
	Special care to be taken with laparoscopic and robotic procedures	Avoid long desiccation times
	Consider local anaesthesia where possible to minimise AGPs	Closed smoke evacuation/filtration system with ULPA capability
	COVID testing for any at-risk patient prior to surgery according to local guidelines and availability	Laparoscopic suction to remove smoke and deflate abdomen
	Most surgery is priority level 4 and can be deferred over 3 months	Low intra-abdominal pressure 10–12 mmHg if feasible
		Avoid rapid deflation
		Minimise blood/fluid droplet spread
		Be careful at time of instrument exchange and tissue extraction
		Minimise CO_2_ leakage from trocars
[27]		Endoscopic and robotic surgery: low electrocautery settings to generate less smoke, lowest pressure insufflation, only essential staff present in theatre, all staff in PPE
[20]	Route of surgery at surgeon’s discretion	Use closed system for insufflation
		Smoke extractor
		Adequate PPE
		Use lowest intrabdominal pressure possible
		Use lowest cautery setting possible
[8]	Symptom screen and COVID test all patients preoperatively	Shorter hospital stay
	Clean COVID-free sites for surgery	Can physical distance more than in open surgery
	All elective surgery for benign indications suspended	Risk of COVID transmission if not operating on GI tract during laparoscopy is low
		Low power diathermy. Closed smoke evacuation
		Filtration system
		Use suction to deflate abdomen
		Low pressure 10–12 mmHg intraoperatively
		Avoid rapid desufflation, minimise blood or fluid spray
		Check seals around all reusable ports
		GA in negative pressure room
[14]	Experienced surgeon to minimise operating time	Filter system to reduce viral release with gas
	Clinical trials and trials of new technology to be postponed	Low pressure pneumoperitoneum
	COVID test all patients preoperatively	Low bipolar cautery setting
	Temperature testing and wearing masks on arrival	
	Reduce inpatient beds to allow physical distancing	
[4]	Suspend all elective surgery	Low intra-abdominal pressure 10–12 mmHg
	Universal COVID-19 testing recommended before all surgery	Low power settings for electrosurgical devices
	Preoperative screening on day of surgery, i.e. history examination	Avoidance of long desiccation times
	Full PPE in theatre—shoe covers, impermeable gowns, surgical or N-95 masks, protective head covering, gloves and eye protection	Closed smoke evacuation or filtration system with ultra-low particulate air filtration capability
	Restricted movement of personnel in and out of the operating room	Suction desufflation of abdomen
	Trainee participation should be limited and include only essential personnel	Avoid rapid desufflation, i.e. with specimen removal
		Minimise CO_2_ leakage from trocars
		Minimise blood/fluid droplet spray
		Vaginal and open surgery: non-electrosurgical techniques where possible
		Low power setting, avoidance of long desiccation times
		Smoke evacuators alongside ULPA filters
		Suction device to remove surgical plume
		Minimize blood/fluid droplet spray or spread
[16]	Enhanced recovery	Limit intra-abdominal pressure
		Balloon trocars to minimize CO_2_ leak
		Smoke extractors
		Suction of CO_2_ for desufflation
[30]	Only urgent procedures to minimise inpatient stays	Safety of minimally invasive surgery remains undetermined
	Screening consultation prior to procedure—symptoms in last 2 weeks, any travel	
	Test patients and clinical team prior to procedure	
	Positive pressure on hold during procedure and restarted 20 min after patient leaves	
	Limited personnel in theatre	
[5]	Recommend only high priority/emergency cases, experienced surgeon	Low insufflation pressure
	Minimal staff numbers, no observers	Suction of gas prior to removing ports
	Intubation and extubation in negative pressure room	Smoke evacuation system capable of filtering aerosolized particles from CO_2_ should be provided for laparoscopic surgery
	Use low cautery settings	
	Avoid monopolar or advanced bipolar where possible	
	If monopolar use smoke evacuator	
	No clear evidence to favour open or laparoscopic	
	Consider treating intermediate priority patients if capacity available but not during COVID surge	
	Follow local recommendations to test staff and patients for COVID	
	Follow local recommendations for PPE	
	Wear full PPE for COVID-positive patients as per WHO guidance	
[31]	All invasive procedures under GA deferred	
[19]	All elective cases deferred	
	Aim for same-day discharge where possible	
	Spinal anaesthesia in preference to general anaesthesia, unlikely to greatly increase voiding dysfunction	
[25]	Transmission of fomites during vaginal surgery appears highly unlikely	
	Regional anaesthesia preferable to general anaesthesia—lower risk postoperative retention, reduces aerosol generation	
[36]	Augmentation cystoplasty, cystectomy, and continent and incontinent diversions all postponed owing to high-dependency in-patient care required	
[21]	Screen all patients with health questionnaire	
	Swab test before all elective surgery	
	All team members trained in appropriate use of PPE	
	Reduce all team members in theatre	
	If COVID positive, operate once fully recovered, i.e. asymptomatic and two negative tests at 24-h interval	
[32]	Ideal is virtual consultation with electronic consent including pre-printed information and patient’s electronic signature	
	Requires development with GMC and MHRA	
	Consent signed on day of procedure may lead to inadequate consent and litigation	

*RCOG* Royal College of Obstetricians and Gynaecologists, *BSGE* British Society for Gynaecological Endoscopy, *EAU* European Association of Urology, *PPE* personal protection equipment, *GA* general anaesthesia, *AGP* aerosol-generating procedure, *GMC* General Medical Council, *MHRA* Medicines and Healthcare Products Regulatory Authority, *ULPA* ultra-low particulate air, *GI* gastrointestinal, *WHO* World Health Organisation

Continuing or restarting surgery during the pandemic requires prioritisation of cases, taking into account the severity of the pathology, patient comorbidities and the impact on physical and mental health and quality of life. Seven documents specified prioritisation guidance. See Table 7 for a summary of the prioritisation of surgery.

**Table 7:** Summary of prioritisation of surgery

Reference	Prioritisation of surgery
[28]	Emergency <1 h: life-threatening emergencies
	Urgent <24 h: e.g. haemorrhage after functional urology surgery, urinary retention, unable to place catheter, surgical site or device infection
	Urgent elective <4 weeks: e.g. second stage of SNS, disabling refractory BPS, Botox in high-risk neurogenic bladder patients, urinary diversion in urinary fistula with severe complications
	Elective, intermediate priority, 1–3 months: e.g. Botox in low-risk neurogenic bladder, bladder outlet obstruction due to mesh, removal of vaginally extruded uninfected mesh, prolapse with complications, e.g. retention, hydronephrosis
	Elective, low-priority, >3 months: e.g. refractory OAB, elective SUI surgery, BPS, elective prolapse surgery, urethral diverticulum without complications, uncomplicated neurogenic bladder
[8]	1a: emergency <24 h to save life
	1b: urgent <72 h as life-threatening condition
	2: is required within <4 weeks with expectation of cure
	3: can defer for 10–12 weeks with no predicated negative outcome
	Enhanced recovery pathways: delay any oncology surgery by at least 15 days if COVID-19 symptoms preoperatively
[16]	A: continue, e.g. second-stage neuromodulation, intravesical Botox for neurogenic bladder with risk of high bladder pressure, surgery for grade 4 prolapse with acute renal failure and failed pessary
	B: 1–8 weeks, e.g. refractory OAB and bladder cancer risk factors
	C: delay 8–16 weeks, e.g. intravesical Botox
	D: can be delayed >16 weeks, e.g. stress urinary incontinence surgery
[29]	1: urgent, <1 month—delay could cause major harm, e.g. prolapse beyond hymen with voiding dysfunction or upper renal tract complications
	2: essential elective, <3 months—increased risk of adverse outcomes if delayed for undetermined time period, e.g. prolapse beyond hymen with progressive symptoms, impaired QoL, failed pessaries but no upper renal tract complications
	3: non-essential elective, postpone up to 1 year—not time sensitive, e.g. prolapse beyond hymen with no upper renal tract complications and able to use pessary
	Continence surgery: non-essential elective, unless failure of conservative and progressive symptoms
[21]	Category 1: urgent: within 30 days, potential to deteriorate and become an emergency
	Category 2: semi-urgent: within 60 days, causes pain dysfunction or disability, but unlikely to deteriorate quickly, unlikely to become an emergency
	Category 3: elective: within 365 days, causes pain dysfunction or disability, unlikely to deteriorate quickly, does not have potential to become emergency
	All urogynaecology cases are category 3, should be postponed. Can start in highly symptomatic patients when risk of transmission reduces, depending on local situations
[5]	Low priority: clinical harm very unlikely if postponed for 6 months, e.g. stress or urge incontinence surgery, surgery for urethral diverticula
	Intermediate: clinical harm possible if postponed for 3–4 months but unlikely, e.g. surgical management of patients with urinary retention, intravesical Botox for selected cases of neurogenic bladder
	High priority: clinical harm likely if postponed for over 6 weeks, e.g. cystoscopy for macroscopic haematuria
	Emergency: life-threatening situation and likely to have presented in ED despite pandemic
[18]	
	Tier 1: non-life-threatening illness, low acuity, i.e. SUI surgery, laparoscopic sacrocolpopexy, native tissue transvaginal prolapse surgery, asymptomatic mesh exposure
	Tier 2: non-life-threatening, but potential for near future morbidity or mortality, intermediate acuity, i.e. fistula repair, mesh-related complication, e.g. severe pain/infection
	Tier 3: high potential for near future morbidity or mortality, severe impairment of QoL, high acuity, i.e. prolapse with upper tract obstruction and unable to retain pessary, obstructed voiding after MUS
	Tier 4: emergency surgery
	Each tier has subsets A and B
	Subset B denotes patients with comorbidities that may be deferred until after lower acuity patients

*EAU* European Association of Urology, *BPS* bladder pain syndrome, *OAB* overactive bladder, *SUI* stress urinary incontinence, *ED* emergency department, *MUS* mid-urethral sling, *QoL* Quality of life

### Strengths

We followed a standardised rapid review methodology in order to provide a summary of recommendations and practice guidelines in a timely manner. We performed a comprehensive literature search including published articles, articles in press and association guidelines to ensure that we identified and included all available evidence regarding management of urogynaecology patients during the COVID-19 pandemic.

There is a high degree of consensus regarding the use of virtual clinics, management outpatient procedures, and surgical techniques to minimise horizontal transmission of COVID-19.

However, variations in recommendations exist and are summarised in this review. Therefore, it can be used as a resource to support adjustments in practice as local conditions evolve.

As further evidence emerges, resources change and the pandemic continues, this synthesis of available guidance can be used as a reference for clinicians to guide management.

### Limitations

Given the aim to issue a summary without delay using rapid review methodology, some studies may have been omitted, which is an inherent limitation of rapid reviews. There is susceptibility to bias in streamlining a systematic review process, for example, in choosing studies for inclusion or exclusion and in data extraction, as fewer independent reviewers conduct each step.

Recommendations are predominantly based on expert opinion and, given the rapidly evolving nature of the COVID-19 virus, there is often a lack of robust scientific evidence [8] for clinically relevant questions.

Indeed, the COVID-19 “infodemic” has been described by WHO as an “overabundance of information—some accurate and some not—that occurs during an epidemic” [37].

This is an inherent limitation of all reviews in this area given the unprecedented public health crisis and the epidemiological characteristics of the current pandemic.

As the COVID-19 pandemic continues, and our understanding and resources change, there is high potential for modifications within recommendations and publication of further guidance, which may have already occurred during publication of this rapid review.

## Conclusion

The COVID-19 pandemic has changed the way in which we conduct healthcare and will do so for the foreseeable future. Evidence suggests that a large proportion of urogynaecological conditions might be able to be managed using virtual consultations utilising behavioural measures, lifestyle changes and medical therapy. Outpatient procedures in one-stop clinics to investigate and treat conditions such as refractory OAB can be maximised to avoid inpatient admissions, and to reduce the frequency of visits and the use of general anaesthesia.

Technology is required to maintain and develop the quality of virtual consultations and this is particularly important for remote teaching of clean intermittent self-catheterisation, home trial without catheter, pessary management and triaging symptoms. For those unable to use or without access to the required technology, smaller ad hoc face-to-face clinics with PPE and physical distancing should be considered.

Various healthcare providers and organisations have developed and published guidance for practice, which should always be observed, as it is linked and adapted to local policies, sociodemographic and epidemiological conditions, as well as infrastructures. This review is aimed at providing a wider perspective on practice recommendations that have been published to date and can be adapted or even considered for implementation at local levels.

Although adaptations and provisions are being made to manage urogynaecological conditions, given that the majority of patients are elderly with comorbidities that increase risk of COVID-19 morbidity and mortality, and with most surgical procedures for quality of life, the resumption of elective activity is expected to be slow. Consequently, there is likely to be a significant impact on quality of life within this cohort of patients and the impact of delayed diagnosis and treatment on the trajectory of the disease is yet to be determined.

## Supplementary Information

ESM 1 (DOCX 12 kb)

ESM 2 (DOCX 127 kb)

ESM 3 (DOCX 71 kb)
