# Supplementary material for: Impact of COVID-19 on management of urogynaecology patients: a rapid review of the literature
Source: Int Urogynecol J. 2021 Feb 3;32(10):2631–46. doi: 10.1007/s00192-021-04704-2 (PMC7856854; doi:10.1007/s00192-021-04704-2)
Supplement: Supplementary file 1 — (DOCX 12 kb) [file 192_2021_4704_MOESM1_ESM.docx]

Appendix 1

Search Strategy:

--------------------------------------------------------------------------------

1 COVID-19.mp. [mp=ti, ab, ot, nm, hw, fx, kf, ox, px, rx, ui, an, sy, tn, dm, mf, dv, kw, dq] (134823)

2 SARS-COV2.mp. [mp=ti, ab, ot, nm, hw, fx, kf, ox, px, rx, ui, an, sy, tn, dm, mf, dv, kw, dq] (2115)

3 coronavirus.mp. [mp=ti, ab, ot, nm, hw, fx, kf, ox, px, rx, ui, an, sy, tn, dm, mf, dv, kw, dq] (158316)

4 1 or 2 or 3 (184318)

5 incontinence.mp. [mp=ti, ab, ot, nm, hw, fx, kf, ox, px, rx, ui, an, sy, tn, dm, mf, dv, kw, dq] (180164)

6 pelvic organ prolapse.mp. [mp=ti, ab, ot, nm, hw, fx, kf, ox, px, rx, ui, an, sy, tn, dm, mf, dv, kw, dq] (26099)

7 vaginal prolapse.mp. [mp=ti, ab, ot, nm, hw, fx, kf, ox, px, rx, ui, an, sy, tn, dm, mf, dv, kw, dq] (3116)

8 uterine prolapse.mp. [mp=ti, ab, ot, nm, hw, fx, kf, ox, px, rx, ui, an, sy, tn, dm, mf, dv, kw, dq] (8380)

9 cystocele.mp. [mp=ti, ab, ot, nm, hw, fx, kf, ox, px, rx, ui, an, sy, tn, dm, mf, dv, kw, dq] (5309)

10 rectocele.mp. [mp=ti, ab, ot, nm, hw, fx, kf, ox, px, rx, ui, an, sy, tn, dm, mf, dv, kw, dq] (4982)

11 bladder pain.mp. [mp=ti, ab, ot, nm, hw, fx, kf, ox, px, rx, ui, an, sy, tn, dm, mf, dv, kw, dq] (4078)

12 childbirth trauma.mp. [mp=ti, ab, ot, nm, hw, fx, kf, ox, px, rx, ui, an, sy, tn, dm, mf, dv, kw, dq] (116)

13 perineal trauma.mp. [mp=ti, ab, ot, nm, hw, fx, kf, ox, px, rx, ui, an, sy, tn, dm, mf, dv, kw, dq] (2202)

14 perineal lacerations.mp. [mp=ti, ab, ot, nm, hw, fx, kf, ox, px, rx, ui, an, sy, tn, dm, mf, dv, kw, dq] (1015)

15 urogynaecology.mp. [mp=ti, ab, ot, nm, hw, fx, kf, ox, px, rx, ui, an, sy, tn, dm, mf, dv, kw, dq] (1391)

16 urogynecology.mp. [mp=ti, ab, ot, nm, hw, fx, kf, ox, px, rx, ui, an, sy, tn, dm, mf, dv, kw, dq] (3983)

17 overactive bladder.mp. [mp=ti, ab, ot, nm, hw, fx, kf, ox, px, rx, ui, an, sy, tn, dm, mf, dv, kw, dq] (27542)

18 recurrent cystitis.mp. [mp=ti, ab, ot, nm, hw, fx, kf, ox, px, rx, ui, an, sy, tn, dm, mf, dv, kw, dq] (799)

19 recurrent urinary tract infections.mp. [mp=ti, ab, ot, nm, hw, fx, kf, ox, px, rx, ui, an, sy, tn, dm, mf, dv, kw, dq] (5528)

20 5 or 6 or 7 or 8 or 9 or 10 or 11 or 12 or 13 or 14 or 15 or 16 or 17 or 18 or 19 (227697)

21 4 and 20 (51)

22 remove duplicates from 21 (37)
