# Supplementary material for: Impact of COVID-19 on management of urogynaecology patients: a rapid review of the literature
Source: Int Urogynecol J. 2021 Feb 3;32(10):2631–46. doi: 10.1007/s00192-021-04704-2 (PMC7856854; doi:10.1007/s00192-021-04704-2)
Supplement: Supplementary file 2 — (DOCX 127 kb) [file 192_2021_4704_MOESM2_ESM.docx]

Appendix 2 AGREEII
